# Exploring the Hidden Talents of Preterm Infants: Moving Beyond a Risk‐Focused Perspective

**DOI:** 10.1111/apa.70684

**Published:** 2026-07-18

**Authors:** Giulia Bertacchini, Livio Provenzi

**Affiliations:** ^1^ Developmental Psychobiology Lab (dpb lab), Department of Brain and Behavioral Sciences University of Pavia Pavia Italy; ^2^ Developmental Psychobiology Lab (dpb lab) IRCCS Mondino Foundation Pavia Italy

## 1 Born Too Soon

Preterm birth is a major global health concern, because it may disrupt developmental processes and expose infants to stressful conditions [1]. Being admitted to a neonatal intensive care unit (NICU) entails painful procedures, sensory overstimulation and reduced parental contact. These have been investigated through a risk‐oriented perspective that emphasizes deficits as the major outcomes of early life exposure to adverse conditions. These negative consequences include visual deficits, lower cognitive outcomes, slower early processing speed and difficulties in attention, memory and executive functions [2, 3]. As survival rates improve, research has increasingly focused on morbidity and the need for appropriate care. We argue that this requires moving beyond a purely deficit‐centred model and considering the emergence of complex, context‐dependent phenotypes. The *hidden talents* approach [4] provided us with a useful starting point for our exploration.

## 2 The *Hidden Talents* Approach

This approach seeks to map social, cognitive and emotional abilities that often remain undetected within deficit‐focused models. These tend to be selectively enhanced or preserved in individuals who are exposed to harsh and unpredictable environments [4]. Harshness refers to conditions that raise morbidity and mortality risk. Unpredictability refers to how much those conditions vary across time and space [4]. For example, a number of socio‐cognitive enhancements have been observed in high‐risk contexts. They include increased procedural learning, attention‐shifting and working memory. They also include improved performance with regard to ecological stimuli that closely resemble real‐life situations. On a socio‐emotional level, studies have reported greater ability to detect emotions and deception, more empathy and heightened vigilance to negative stimuli [4]. This framework emphasizes that outcomes should be interpreted in light of an individual's environmental context rather than against standardized benchmarks. Drawing on this insight, the concept of adaptation as a fixed outcome inferred from its pragmatical advantage for survival shifts to adapting as an ongoing, within‐lifetime developmental process. This perspective aligns with neuroconstructivism and dynamic systems theory. Both view development as an emergent, context‐dependent process shaped by biological and environmental factors [5].

## 3 Exploring *Hidden Talents* in Preterm Children

Applying the *hidden talents* framework to preterm populations reflects the idea that environmental variations shape specific phenotypes. NICUs are inherently stressful for preterm infants and they provide naturally available test cases [1]. The dimension of harshness is represented by the high morbidity risk and biological stressors, while unpredictability reflects variable sensory and social inputs. To date, this framework has been relatively unexplored in relation to prematurity, highlighting the need to identify population‐specific strengths and appropriate methods of investigation. For example, these skills should be framed as early processing tendencies whose developmental continuity and causal pathways cannot be fully inferred yet. The goal is to generate new evidence by reinterpreting existing findings within a framework of multifinal, heterogeneous developmental trajectories [2, 5] (Table 1).

**TABLE 1:** Summary of main results from research conducted so far highlighting potential *hidden talents* in preterm infants and children.

Study	Domain	Results	Potential *hidden talents*
Fallang et al. (2003) [6]	Sensorimotor integration	More optimal reaching at 4 months	Enhanced basic sensorimotor integration for environmental exploration
Eilers et al. (1993) [7]	Sensorimotor integration and early communication	Earlier canonical babbling with rhythmic hand banging	Faster sensorimotor learning
Hunnius et al. (2008) [8]	Attention	Faster disengagement and shifting in competitive tasks	Rapid detection of changes
Jaeger et al. (2021) [9]	Alertness	Preserved decision‐making in phasic conditions, slower tonic	Quick response to environmental cues
Finke et al. (2015) [10]	Attention	Reduced VSTM, preserved speed, top‐down mechanisms and attention orienting	Procedural learning optimized
Witt et al. (2014) [11]	Emotion recognition	Same level of emotion recognition of full terms with ecological stimuli	Prioritization of socially relevant stimuli over abstract stimuli
Longfier et al. (2016) [12]	Emotional expression and sensory processing	Reduced negative facial expressions to new foods	Early adaptation to unpredictable environments
Hille et al. (2008) [13]	Social‐affective behaviour	Generally lower criminal behaviour, substance use, sexual activity, teenage pregnancy	Prioritization of self‐preservation
Alenius et al. (2023) [14]	Social‐affective behaviour	Generally lower criminal behaviour, substance use, sexual activity, teenage pregnancy	Prioritization of self‐preservation

From a socio‐cognitive perspective, the study of Hunnius et al. [8] shows that preterm infants and full‐term infants behaved differently in a visual attention task. In the non‐competition trial, a central stimulus disappeared before a peripheral one appeared. In the competition trial, the central stimulus persisted even after the peripheral target had appeared. Up to 16 weeks post‐term, preterm infants disengaged attention from the initial stimulus faster than full‐term infants during the competitive task. Interestingly, this ability did not seem to develop because of greater visual experience. It seemed to be the result of an acceleration of cortical functions, which indicated specific neural reorganization. Jaeger et al. [9] investigated tonic and phasic alertness in 5‐year‐old children born preterm. Tonic alertness is sustained readiness, measured here with randomly timed targets. Phasic alertness is a brief arousal spike triggered, in this case, by an auditory cue before the target. In the tonic condition, preterm children were slower across all measures. In the phasic condition, reaction and decision‐making times normalized, but movement times remained slower, suggesting partially independent developmental paths. Finke et al. [10] studied adults who were born preterm and reported that visual attention deficits were mainly due to reduced storage capacity of visual short‐term memory. In contrast, processing speed, basic spatial orientation and top‐down mechanisms were relatively preserved. Interestingly, *hidden talents* theory suggests that working memory reductions may be trade‐offs favouring associative learning and automatic responses under stress. Since visual short‐term memory storage represents a core component of working memory, future research should explore whether similar trade‐offs apply here [4]. Another notable finding of the study of Finke et al. [10] was that functional magnetic resonance imaging showed different connectivity in the parietal and occipital cortices. Greater reorganization in these cortices resulted in greater preservation of this function, highlighting a possible atypical developmental trajectory [10]. Witt et al. [11] investigated the socio‐emotional domain of preterm children at 42 months of age and compared them with children born full‐term. This showed that the children born preterm performed worse on an emotion recognition task where they were asked to identify emotions based on facial expressions. Yet they matched full‐term peers when social context was provided, even if this ability tends to emerge later on in development [11]. This aligns with the *hidden talents* framework, which predicts better performance on ecological tasks [4]. Overall, these findings may have reflected context‐dependent trade‐offs and potentially complex atypical developmental trajectories [8, 10, 11] shaped by stress and biological constraints. However, these findings were not conclusive. More speculative interpretations would rely on further longitudinal investigations that track the phenomenon over time.

## 4 Implications and Future Directions

**FIGURE 1:**
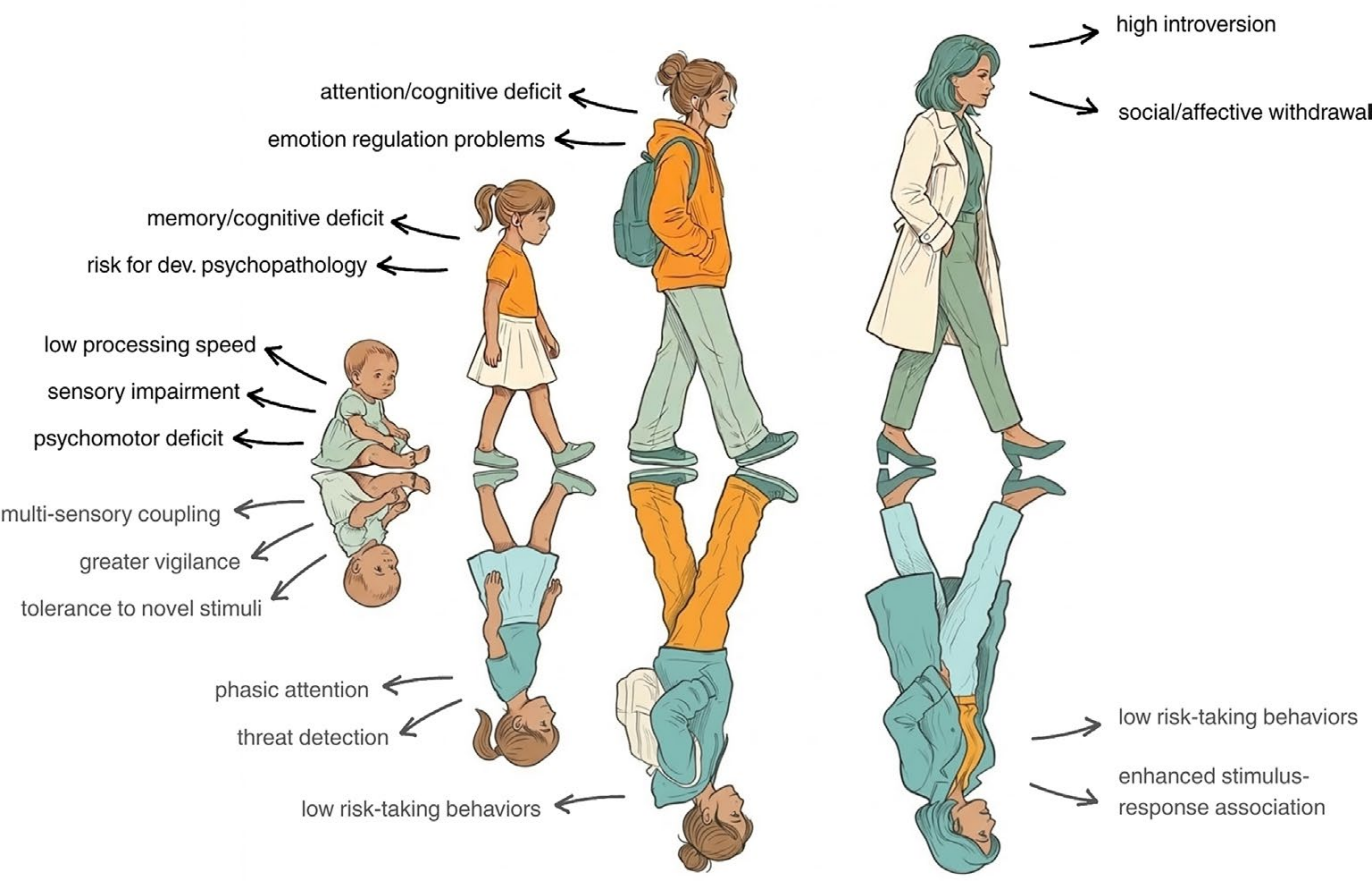
Graphical representation of preterm birth consequences according to the deficit model (upright figures) and to the *hidden talents* approach (mirrored figures).

Our research suggests that the *hidden talents* approach can provide a more comprehensive understanding of preterm‐born children's abilities, helping to leverage them effectively. For example, if these children perform better with ecological stimuli, it might be possible to design more respectful and targeted educational interventions. From a theoretical perspective, future research should consider these abilities as traces of deeper, context‐dependent developmental processes that may act as hidden mechanisms shaping neural reorganization. Neuroscientific techniques, cross‐population and longitudinal studies could help to disentangle the pathways of development of such emerging functions. Although this line of research is promising, it is still in its preliminary stages and has produced correlational findings from small, heterogeneous samples. At the same time, we strongly believe that investing resources and energies in such an approach could encourage clinicians and researchers to walk on less travelled paths. This will enable them to deeply value the complexity in early development that is demonstrated in Figure 1.

## Author Contributions

**Giulia Bertacchini:** conceptualization, writing – original draft. **Livio Provenzi:** conceptualization, writing – review and editing, supervision.

## Funding

This work was supported by Ministero della Salute (GR‐2021‐12375213, Project 2‐BRAINED) and Ministero dell'Università e della Ricerca (National Recovery and Resilience Plan, NRRP; project MNESYS, PE0000006 – A Multiscale integrated approach to the study of the nervous system in health and disease, DN. 1553 11.10.2022).

## Conflicts of Interest

The authors declare no conflicts of interest.

## Data Availability

Data sharing is not applicable to this article as no datasets were generated or analysed during this study.

## References

[1] J. Santos , S. E. Pearce , and A. Stroustrup , “Impact of Hospital‐Based Environmental Exposures on Neurodevelopmental Outcomes of Preterm Infants,” Current Opinion in Pediatrics 27, no. 2 (2015): 254–260, 10.1097/MOP.0000000000000190. 25635585 PMC4410011

[2] P. J. Anderson , “Neuropsychological Outcomes of Children Born Very Preterm,” Seminars in Fetal and Neonatal Medicine 19, no. 2 (2014): 90–96, 10.1016/j.siny.2013.11.012. 24361279

[3] D. DeMaster , J. Bick , U. Johnson , J. J. Montroy , S. Landry , and A. F. Duncan , “Nurturing the Preterm Infant Brain: Leveraging Neuroplasticity to Improve Neurobehavioral Outcomes,” Pediatric Research 85 (2019): 166–175, 10.1038/s41390-018-0203-9. 30531968

[4] B. J. Ellis , L. S. Abrams , A. S. Masten , R. J. Sternberg , N. Tottenham , and W. E. Frankenhuis , “Hidden Talents in Harsh Environments,” Development and Psychopathology 34, no. 1 (2022): 95–113, 10.1017/S0954579420000887. 32672144

[5] A. Karmiloff‐Smith , “Nativism Versus Neuroconstructivism: Rethinking the Study of Developmental Disorders,” Developmental Psychology 45, no. 1 (2009): 56–63, 10.1037/a0014506. 19209990

[6] B. Fallang , O. D. Saugstad , J. Grøgaard , and M. Hadders‐Algra , “Kinematic Quality of Reaching Movements in Preterm Infants,” Pediatric Research 53, no. 5 (2003): 836–842, 10.1203/01.PDR.0000058925.94994.BC. 12612201

[7] R. E. Eilers , D. K. Oller , S. Levine , D. Basinger , M. P. Lynch , and R. Urbano , “The Role of Prematurity and Socioeconomic Status in the Onset of Canonical Babbling in Infants,” Infant Behavior and Development 16, no. 3 (1993): 297–315, 10.1016/0163-6383(93)80037-9.

[8] S. Hunnius , R. H. Geuze , M. J. Zweens , and A. F. Bos , “Effects of Preterm Experience on the Developing Visual System: A Longitudinal Study of Shifts of Attention and Gaze in Early Infancy,” Developmental Neuropsychology 33, no. 4 (2008): 521–535, 10.1080/87565640802101508. 18568902

[9] D. A. Jaeger , N. Gawehn , D. T. Schneider , and B. Suchan , “Phasic and Tonic Alertness in Preterm 5‐Year‐Old Healthy Children,” Child Neuropsychology 27, no. 8 (2021): 1073–1087, 10.1080/09297049.2021.1919297. 33899687

[10] K. Finke , J. Neitzel , J. G. Bäuml , et al., “Visual Attention in Preterm Born Adults: Specifically Impaired Attentional Sub‐Mechanisms That Link With Altered Intrinsic Brain Networks in a Compensation‐Like Mode,” NeuroImage 107 (2015): 95–106, 10.1016/j.neuroimage.2014.11.062. 25498391

[11] A. Witt , A. Theurel , C. B. Tolsa , et al., “Emotional and Effortful Control Abilities in 42‐Month‐Old Very Preterm and Full‐Term Children,” Early Human Development 90, no. 10 (2014): 565–569, 10.1016/j.earlhumdev.2014.07.008. 25105752

[12] L. Longfier , R. Soussignan , N. Reissland , et al., “Emotional Expressiveness of 5‐6 Month‐Old Infants Born Very Premature Versus Full‐Term at Initial Exposure to Weaning Foods,” Appetite 107 (2016): 494–500, 10.1016/j.appet.2016.08.124. 27593453

[13] E. T. Hille , C. Dorrepaal , R. Perenboom , J. B. Gravenhorst , R. Brand , and S. P. Verloove‐Vanhorick , “Social Lifestyle, Risk‐Taking Behavior, and Psychopathology in Young Adults Born Very Preterm or With a Very Low Birthweight,” Journal of Pediatrics 152, no. 6 (2008): 793–800, 10.1016/j.jpeds.2007.11.041. 18492518

[14] S. Alenius , E. Kajantie , R. Sund , et al., “Risk‐Taking Behavior of Adolescents and Young Adults Born Preterm,” Journal of Pediatrics 253 (2023): 135–143.e6, 10.1016/j.jpeds.2022.09.032. 36179892

